# microRNA-125b inhibits cell migration and invasion by targeting matrix metallopeptidase 13 in bladder cancer

**DOI:** 10.3892/ol.2022.13509

**Published:** 2022-09-20

**Authors:** Deyao Wu, Jingjing Ding, Linmao Wang, Huixing Pan, Zhengdong Zhou, Jian Zhou, Ping Qu

Oncol Lett 5: 829–834, 2013; DOI: 10.3892/ol.2013.1123

Following the publication of the above article, it was drawn to the Editors’ attention by a concerned reader that several of the panels showing the results from cell migration and invasion assay experiments in Fig. 2 on p. 831 contained sections of data that were overlapping with data presented in the same figure, occasionally in different orientations; furthermore, some of the data were also included in another article published at around the same time featuring some of the same authors. Given the issues of the overlapping data sections and the re-use of data in another article, the Editor of *Oncology Letters* has determined that this paper should be retracted from the Journal on account of the sharing of data and a lack of confidence in the presented results. The authors were asked for an explanation to account for these concerns, but the Editorial Office did not receive a satisfactory reply. The Editor apologizes to the readership for any inconvenience caused.

